# Adsorption of Scandium Ions by Amberlite XAD7HP Polymeric Adsorbent Loaded with Tri-n-Octylphosphine Oxide

**DOI:** 10.3390/molecules29071578

**Published:** 2024-04-01

**Authors:** Diana Daminescu, Narcis Duteanu, Mihaela Ciopec, Adina Negrea, Petru Negrea, Nicoleta Sorina Nemeş, Bogdan Pascu, Cătălin Ianăşi, Lucian Cotet

**Affiliations:** 1 Faculty of Industrial Chemistry and Environmental Engineering, Politehnica University of Timisoara, Victoriei Square, No. 2, 300006 Timisoara, Romania; 2 Renewable Energy Research Institute-ICER, Politehnica University of Timisoara, Gavril Musicescu Street, No. 138, 300774 Timisoara, Romania; ioan.pascu@upt.ro; 3 Coriolan Drăgulescu’ Institute of Chemistry, Bv. Mihai Viteazul, No. 24, 300223 Timisoara, Romania; 4 Alum, S.A, Isaccei Street No. 83, 820228 Tulcea, Romania; lcotet@alum.ro

**Keywords:** scandium, recovery process, adsorption, Amberlite XAD7HP, TOPO

## Abstract

In an actual economic context, the demand for scandium has grown due to its applications in top technologies. However, further development of new technologies will lead to an increase in the market for Sc related to such technologies. The present study aims to improve and upgrade existing technology in terms of efficient scandium recovery, proposing a new material with selective adsorptive properties for scandium recovery. To highlight the impregnation of Amberlite XAD7HP resin with tri-n-octylphosphine oxide extractant by the solvent-impregnated resin method, the obtained adsorbent material was characterized by physico-chemical techniques. Further, the specific surface of the adsorbent and the zero-point charge of the adsorbent surface have been determined. Different parameters, such as initial concentration, adsorbent amount, contact time, or temperature, have been studied. The initial pH effect was investigated when a maximum adsorption capacity of 31.84 mg g^−1^ was obtained at pH > 3, using 0.1 g of adsorbent and a contact time of 90 min and 298 K. An attempt was made to discuss and provide a clear representation of the studied adsorption process, proposing a specific mechanism for Sc(III) recovery from aqueous solutions through kinetic, thermodynamic, and equilibrium studies. Adsorption/desorption studies reveal that the prepared adsorbent material can be reused five times.

## 1. Introduction

One of the rare earth elements (REE) having an important role in the development of new technologies is Sc. This element presents distinct chemical properties, some of which are similar to those of yttrium and lanthanum. Due to his distinct mechanical and chemical properties, Sc is used in a wide range of applications, including aviation, automotive, fuel cell technologies, laser diodes, and many other industrial applications [1,2]. In this context, it can be said that Sc represents a valuable element, but one must consider that this element is less widespread in nature than other natural minerals. Regularly, Sc could be extracted as a by-product from different industrial wastes, e.g., red mud tailing obtained during bauxite production, along with a huge number of other elements. In addition to these other elements, an important problem is represented by the presence of iron and aluminum ions due to their similar properties with Sc ions, which pose some separation problems [3].

Therefore, in these conditions, recovery from different industrial wastes, even if it is desirable, represents a big challenge [4]. A large number of experimental studies were carried out in order to streamline Sc ions recovery and separation from the leaching liquid of various raw ore. These separation methods involved liquid membrane separation, ion exchange, solvent extraction [5], adsorption, or co-precipitation [6]. The most efficient method among them is represented by the solvent extraction method, but this method is inconvenient due to the higher toxicity of the involved chemicals [5]. Different techniques were used in hydrometallurgy for the recovery of different metals from ores or waste solutions, each of which had its own advantages and disadvantages. During time, different extraction techniques were developed, such as solvent extraction, supported liquid membrane techniques, solid-liquid extraction, ion exchange with chelating resins, and impregnated ones [6,7,8,9,10]. Another group of techniques involved in Sc recovery is represented by solid-phase extraction involving functionalized macroligands on a stationary phase [11]. Another technique used for the recovery of different valuable metallic ions is the liquid-phase polymer-based retention (LPR) technique [12]. Drawing from these techniques, a new adsorptive process named polymer enhanced ultrafiltration (PEUF) was developed by coupling ultrafiltration with adsorption on water-soluble polymers [13]. The continuous increase in the need for raw materials boosted the development of new technologies used for metallic ion recovery. One of these new technologies is represented by the use of thermosensitive polymers, which are water-soluble at low temperatures when they bond metallic ions. Further, as temperature increases, they become non-soluble at the lower critical solution temperature, allowing the separation of desired metallic ions [14,15].

From all these technologies, the most studied one was organic solvent extraction, which represents a common separation technology for metallic ions [10]. The selection of proper organic extractants and aqueous solutions is influenced by a large variety of criteria, including performance, environmental consequences, and, in the end, economic considerations [16]. During the last decades, organic chemical compounds have been used as extractants in various hydrometallurgical processes, either alone or in synergistic mixtures, to achieve successful separation of the desired metallic ions. Solvent extraction and solid-phase extraction represent two techniques that are being increasingly used for the selective separation of different metallic ions [17]. From all the techniques developed for metallic ion recovery, the most eloquent one is adsorption, both from the point of view of the efficiency of the metallic ion recovery and from the economic point of view. In the specialized literature are presented a multitude of materials with adsorbent properties used to remove toxic metallic ions from water, such as metal oxides/hydroxides [18,19,20,21], zeolites [22,23,24], red mud [25], dolomite [26], commercial or synthetic activated carbon [27,28], biomass [29], activated alumina [30,31,32], bone meal [33], biopolymers [34], etc.

Currently, researchers have prepared new classes of adsorbent materials used for further recovery and selective separation of different metallic ions from aqueous solutions. In this sense, it was impetuously necessary to improve their adsorption properties. In order to improve adsorbent properties, it was necessary to develop new methods for chemical modification through functionalization with different functional groups containing nitrogen, phosphorus, and sulfur. During the last decades, special attention was paid to the usage of different supports: inorganic solid supports (silica, magnesium silicate, etc.) or polymeric ones such as aromatic copolymers such as styrene—divinylbenzene (XAD-2, XAD-4, XAD-12, XAD-16, XAD-1180, XAD-2000, XAD-2010), aliphatic ester-type copolymers (XAD-7, etc.), acrylic ester-type copolymers (XAD-8, etc.). In the same period, they were used as supports for aromatic compounds functionalized with organophosphoric acids and chelated copolymers with phosphonic and phosphinic groups [34,35,36,37,38,39,40,41,42,43,44]. Other materials presented in the literature as materials with good adsorbent properties are different natural polymers, the best known of which are chitosan [45] and cellulose [46,47,48,49]. Taking all these into account, studies were performed in order to prepare new adsorbent materials through physical modification (impregnation) in order to improve adsorbent properties, which were further used in order to selectively recover Sc(III) ions from aqueous medium. Thus, this work presents a new approach in terms of synthesis, characterization, and further application of the material obtained by functionalization by impregnation.

Adsorbent functionalization by impregnation of solid adsorbent was achieved by using the solvent impregnated resin (SIR) method, allowing us to produce alternative and effective adsorbent materials for various metallic ion recovery processes. The SIR method allows functionalization of different solid supports (resins or ion exchangers, silica, and magnesium silicate) with different extractants containing different pendant groups (N, P, S, -COOH, and -CHO). Among the known extractants we mention are di(2-ethylhexyl) phosphoric acid (DEHPA), tri-n-octylphosphine oxide (TOPO), tri-phenyl-phosphine oxide (TPPO), crown ether (dibenzo-18-crown-6), and a mixture of organophosphoric acid extractants.

Thus, the present study describes the absorptive and selective behavior of a newly prepared XAD7HP-TOPO adsorbent material produced by functionalizing XAD7HP Amberlite resin with TOPO extractant for Sc(III) ion recovery from aqueous solutions.

## 2. Results and Discussion

### 2.1. Characterization of Adsorbent

**Figure 1:**
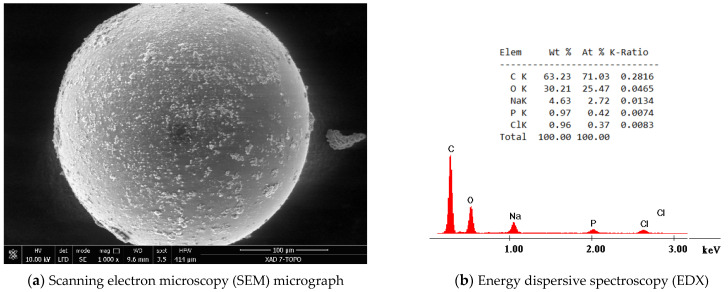

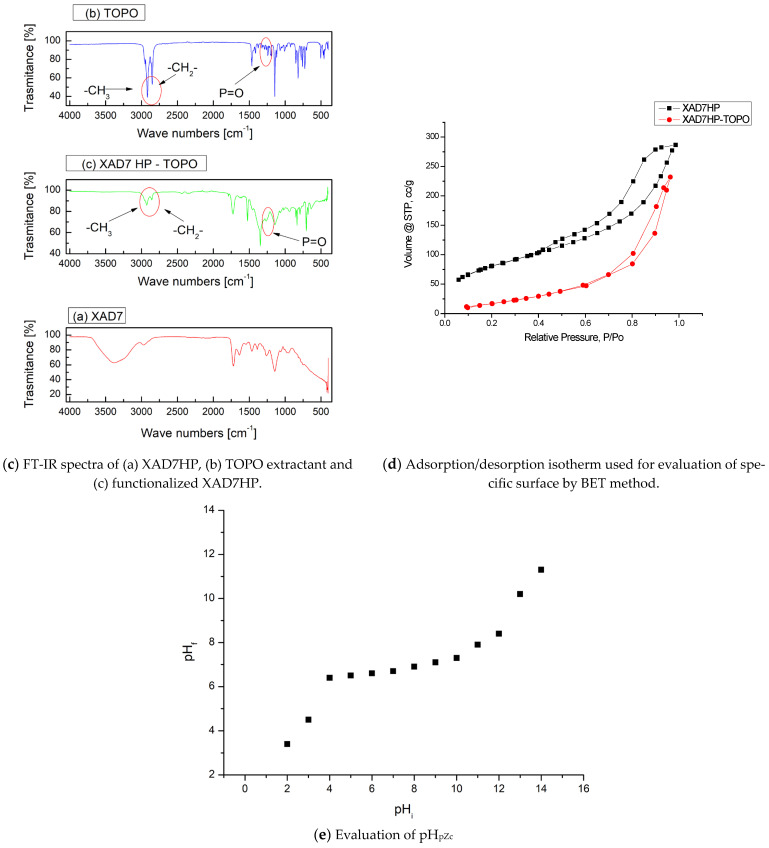
Material characterization.

After adsorbent material preparation, his physico-chemical characterization was performed in order to prove that the Amberlite XAD7HP solid support was functionalized with TOPO extractant. So, SEM (micrograph depicted in Figure 1a), EDX (EDX spectra depicted in Figure 1b), and FT-IR (spectra depicted in Figure 1c) characterized functionalized adsorbent material. At the same time, the specific surface before and after functionalization was evaluated (adsorption/desorption isotherms are depicted in Figure 1d). Further, the value of pHpzc was determined (data depicted in Figure 1e).

SEM analysis has been performed in order to identify the shape and further the morphology of the outer surface of the XAD7HP functionalized with TOPO extractant. As can be seen from the micrograph depicted in Figure 1a, the XAD7HP resin surface becomes cloudy after impregnation. Such color changes indicate a successful impregnation of the XAD7HP resin. Also, from the recorded micrograph, we can observe that after resin impregnation, his surface morphology has been affected through functionalization (the presence of the small micro-granules fixed onto the resin granules). The presence of these micro-granules fixed to the surface of the XAD7HP resin indicates its functionalization with TOPO extractant.

Analyzing the EDX spectra presented in Figure 1b, we can observe the presence of a peak (P peak) associated with the presence of TOPO extractant on the surface of Amberlite. From the FT-IR spectra (Figure 1c), it is observed that there is the presence of an adsorption peak located at 1300 cm^−1^, a peak associated with the vibration of P=O groups. Peaks located at 2995 and 2923 cm^−1^ are associated with the stretching vibrations of –CH_3_ and –CH_2_ groups embedded into the TOPO molecule fixed onto the XAD7HP surface [50].

Analyzing the adsorption/desorption isotherm depicted in Figure 1d, we can observe that the adsorption isotherm recorded for the support material (Amberlite XAD7HP) is a type IV(a) with a type H2(b) hysteresis one. The type H2(b) hysteresis is associated with pore blocking, but the size distribution of the pore neck widths is much larger. Based on the recorded adsorption isotherm, it was determined that the unfunctionalized material has a specific surface area of 520 m^2^ g^−1^. Further, we recorded the adsorption/desorption isotherm for the newly prepared adsorbent material. From the data presented in Figure 1d, we can also observe that in the case of XAD7HP-TOPO (prepared adsorbent material), the recorded adsorption isotherm is a type IV(a) one with type H3 hysteresis. Such hysteresis types are given by non-rigid aggregates of particles, but also if the network pores consist of macropores that are not completely filled with pores condensate [51]. As a consequence of support functionalization, the specific surface of Amberlite support decreases to 52 m^2^ g^−1^ due to the presence of TOPO molecules on the support surface/pores.

From the pH_f_ vs. pHi dependence, it was determined that the newly prepared adsorbent material has a pHpzc of 6.5. At a pH higher than pHpzc, the surface of the adsorbent material is negatively charged (dissociation of different carboxyl groups occurs, and in this way, the cationic species can be retained). At pH values lower than pHpzc, the surface of the adsorbent material is positively charged, superficial hydroxyl groups fix the protons, and the system has a high affinity for anions. Taking into account the pHpzc calculated value, it can be stated that the retention of Sc(III) ions in acidic media is possible only in the anionic form Sc(OH)_4_^−^ [52]. This conclusion is in concordance with the data obtained from the study regarding the influence of the pH over the maximum quantity of retained Sc ions.

### 2.2. Sc(III) Adsorption Kinetics

Sc(III) adsorption kinetic studies were carried out starting from the variation of contact time and temperature, keeping constant the initial concentration of Sc(III) ions, the dose of the adsorbent material, and the pH of the solution.

#### Influence of Contact Time and Temperature

**Figure 2:**
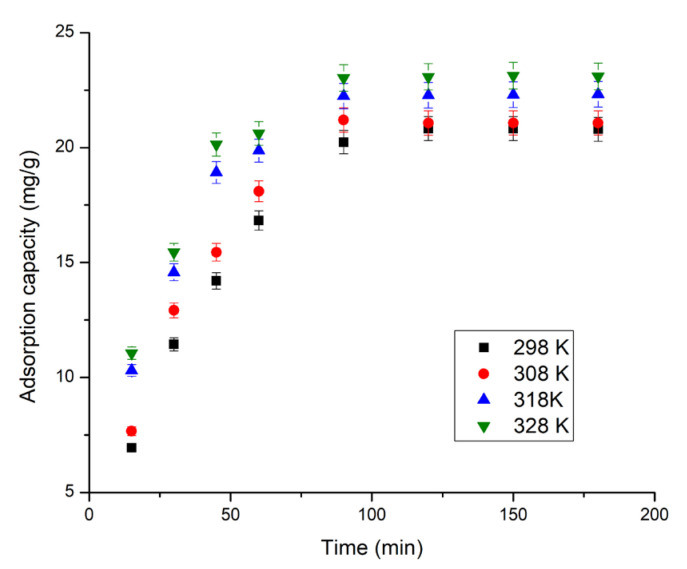
Effect of contact time and temperature (contact time ranged between 15 and 120 min, adsorbent amount 0.1 g, solution volume 25 mL, Sc(III) initial concentration 100 mg L^−1^).

The contact time was one of the studied parameters and through its variation in the range between 15 and 120 min, the obtained data are depicted in Figure 2. Studies regarding the influence of contact time were carried out at four different temperatures (298, 308, 318, and 328 K). From the data presented in Figure 2, it can be observed that with the increase in contact time, the adsorption capacity of the XAD7HP-TOPO adsorbent material increases, reaching a maximum value after 90 min, which is considered the equilibrium time for Sc(III) ion adsorption.

Another parameter that can positively influence adsorptive processes is temperature. It was expected that higher temperatures would facilitate the adsorption of the metallic ions because they would increase their mobility, which would lead to more efficient interaction with functional groups of the adsorbent material. Data obtained for Sc(III) adsorption on XAD7HP-TOPO are depicted in Figure 2. Analyzing these data, it can be observed that with the increase in temperature, the adsorption capacity of the adsorbent material increases as expected (from 20.24 mg g^−1^ at 298 K to 23.03 mg g^−1^ at 238K). From economic consideration and because the increase in adsorption capacity was insignificant, any further studies were carried out at 298 K.

**Figure 3:**
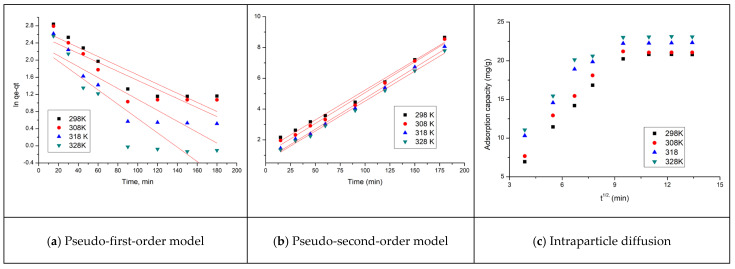
Kinetics model and intraparticle diffusion.

Such kinetic studies are important to establish the mechanism of the adsorption process, especially the reaction speed of the process, but also the way the adsorption process proceeds, by intraparticle diffusion or film diffusion. In order to evaluate the reaction speed obtained, experimental data were modeled using the pseudo-first-order (Lagergren model—data depicted in Figure 3a) and pseudo-second-order (Ho and McKay—data depicted in Figure 3b) models. Depending on the value of the *R*^2^ coefficient obtained after modeling the experimental data, the model that better described the obtained experimental data was chosen. A better way to confirm if the used model fits the obtained experimental data is by using the adjusted R squared coefficient. Mathematical statistics demonstrate that the models with a higher R squared value and a lower adjusted R squared do not better describe the experimental data [53,54]. Thus, in the studied case, the pseudo-second-order kinetic model is the one that best describes the experimental data regarding Sc(III) ion adsorption. Parameters associated with the model used for kinetic studies are presented in Table 1.

The main mechanism involved in Sc(III) ion adsorption is the formation of chemical bonds between active centers located on the surface of the adsorbent material and the Sc(III) ionic species presented in the aqueous medium. Applying the intraparticle diffusion model (Weber and Morris) to the experimental data obtained for Sc(III) ion adsorption highlights the fact that the studied process takes place in two distinct stages, enlisting a much more pronounced effect for high concentrations of Sc(III) ions. In the first stage, Sc(III) ions quickly occupy the free active centers founded on the adsorbent material surface through external diffusion, and in the second stage, these ions enter the adsorbent particles by intraparticle diffusion. This second process takes place until the equilibrium state is reached.

### 2.3. Thermodynamic Studies

#### 2.3.1. pH Influence

**Figure 4:**
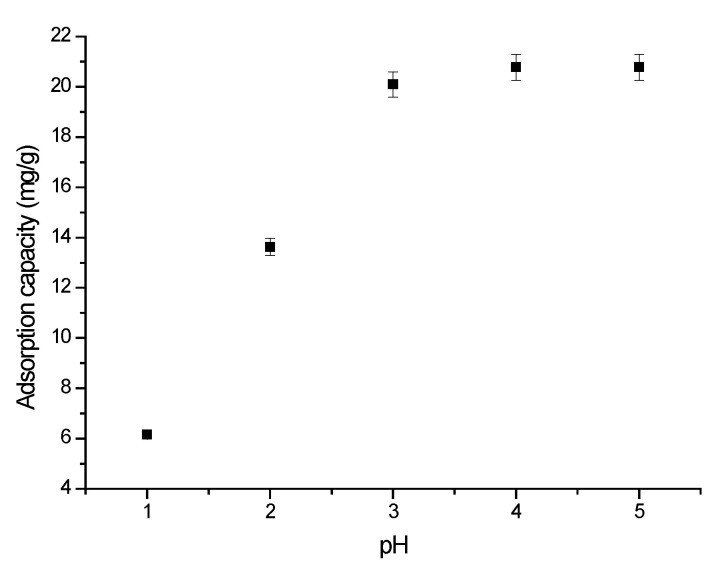
Effect of Sc(III) solution initial pH (pH ranged between 1 and 5, 0.1 g of adsorbent material, contact time 60 min, 298 K, Sc(III) initial concentration 100 mg L^−1^).

An important parameter for all adsorptive processes is represented by the initial pH of the solution. This parameter influences the ionic state of the Sc ions and influences the chelating agent in the solution [55]. pH influence over Sc(III) ion adsorption was studied in the pH range between 1 and 5, with the obtained data being depicted in Figure 4. Analyzing the obtained data, we can observe that the adsorption capacity has a sharp increase with the pH increase until the pH reaches 3. Sc(III) adsorption capacity remains constant for any further increase in the pH value. Based on this observation, any further experiments regarding Sc(III) adsorption on XAD7HP-TOPO were carried out at a pH higher than 3.

#### 2.3.2. Influence of Sorbent Amount

**Figure 5:**
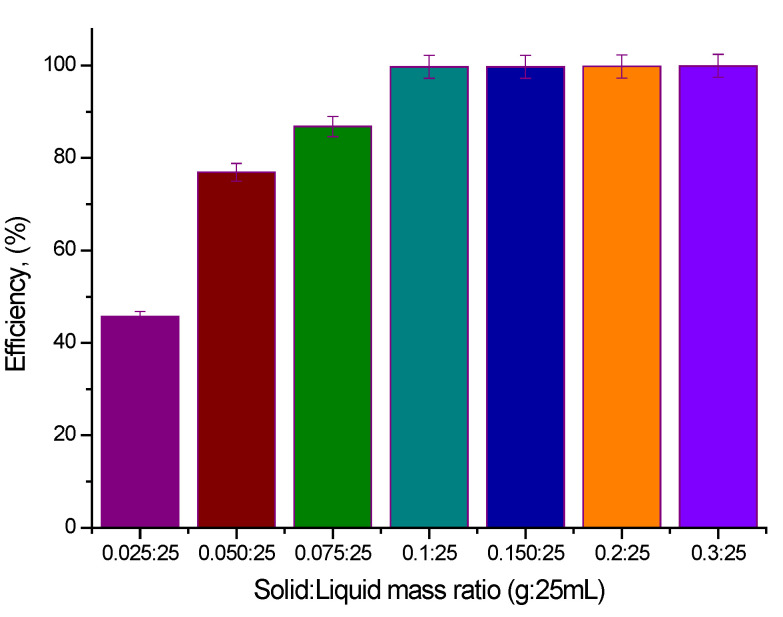
Effect of adsorbent amount (adsorbent amount ranged between 0.025 and 0.3 g, solution volume 25 mL, Sc(III) initial concentration 100 mg L^−1^, contact time 90 min).

Another specific parameter for adsorption processes is represented by the adsorbent amount. In the present study, the influence of this parameter on Sc(III) adsorption was determined by varying the adsorbent amount between 0.025 and 0.3 g and keeping constant the volume of solution (25 mL) and the Sc(III) initial concentration (100 mg Sc(III) per L). Based on the data presented in Figure 5, we can observe that the efficiency of the studied process increased considerably until the ratio of solid:liquid = 0.1 g: 25 mL (a maximum efficiency of 99.7%). For any further increase in the ratio of solid:liquid, the efficiency of the adsorptive process remains constant.

#### 2.3.3. Influence of Sc(III) Ions Initial Concentration and Adsorption Isotherms

**Figure 6:**
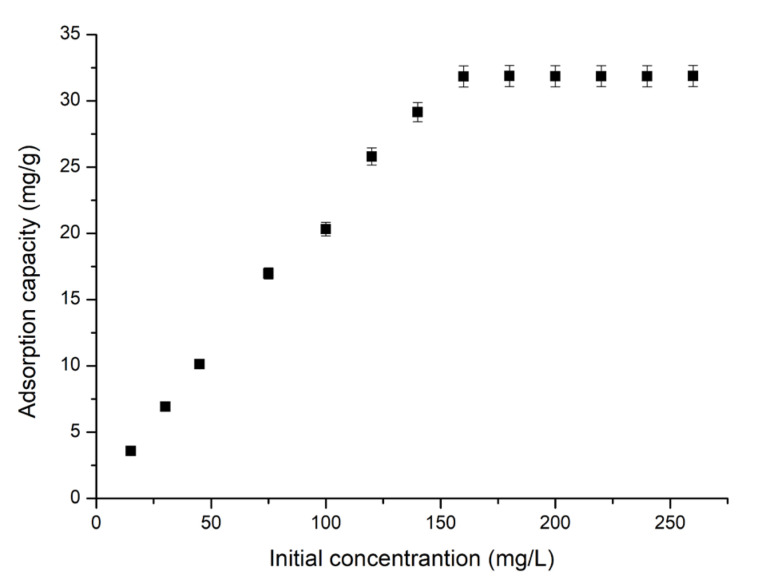
Effect of initial concentration (Sc(III) initial concentration ranged between 15 and 200 mg L^−1^, adsorbent amount 0.1 g, solution volume 25 mL, contact time 90 min).

It is well known that the initial concentration of Sc(III) ions is very important for establishing the equilibrium condition for the studied adsorption process. In this context, Sc(III) concentration was varied into the range of 15 to 200 mg Sc(III) ions per L, resulting in the experimental data presented in Figure 6. From these data, we can conclude that the maximum adsorption capacity of 31.84 mg g^−1^ was obtained in the case of using an initial concentration of 160 mg L^−1^.

**Figure 7:**
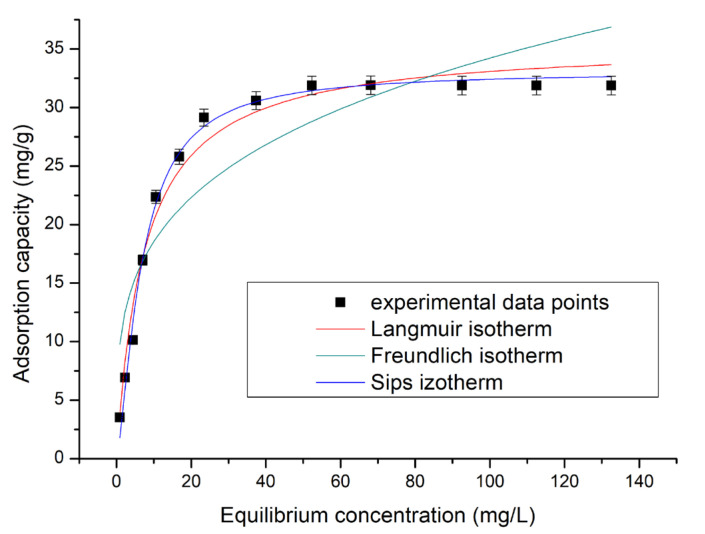
Adsorption isotherms obtained for Sc(III) adsorption onto XAS7HP-TOPO.

The functional dependence between the adsorbate concentration fixed onto the surface of the adsorbent surface and the adsorbate concentration in the liquid phase at equilibrium and constant temperature is represented by adsorption isotherms (the obtained data are depicted in Figure 7). The data represented in Figure 7 were obtained by modeling experimental data with three isotherms (Langmuir, Freundlich, and Sips). By applying these models to the obtained experimental data, the specific parameters for each adsorption isotherm concomitant with correlation parameters were determined (the data are presented in Table 2).

By analyzing the data presented in Table 2, we can observe that the value of the determination coefficient (*R*^2^) is closer to unity when the experimental data were modeled by the Sips isotherm. Also, it can be observed that the calculated adsorption capacity in this case has a value of 34.6 mg g^−1^, being closest to the experimental one (31.84 mg g^−1^). Taking into account these two observations, we can conclude that the Sc(III) adsorption on XAD7HP-TOPO is better described by the Sips adsorption isotherm.

Below (Table 3) are some examples of adsorbent materials with similar properties used for Sc(III) ion recovery from different solutions by adsorption. From the data presented in Table 3, we can observe that the newly prepared material presents the highest Sc(III) recovery efficiency.

#### 2.3.4. Thermodynamic Feasibility of Sc(III) Adsorption, Activation Energy

**Figure 8:**
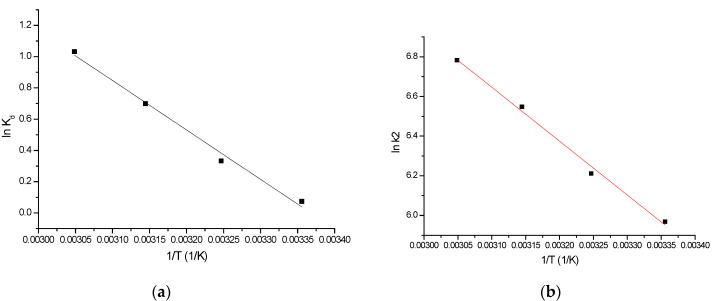
Thermodynamic studies performed for Sc(III) adsorption on XAD7HP-TOPO adsorbent material. (**a**) ln K_d_ = f(1/T), (**b**) ln K_2_ = f(1/T).

In order to determine if the studied process is a physical or a chemical one, thermodynamics was studied. In this context, the van’t Hoff equation (a graphical representation of the linear form of this equation is presented in Figure 8) was used to calculate the associated thermodynamic parameters (the obtained data are presented in Table 4). Further, the value of the activation energy was evaluated from the linear dependence between ln k_2_ vs. 1/T (the obtained graph is presented in Figure 8).

A thermodynamic study reveals that the free Gibbs energy (ΔG^0^) presents negative values that increase with temperature, suggesting that the studied adsorptive process is a spontaneous one. In the meantime, the positive value of the enthalpy (ΔH^0^) confirms the endothermic nature of the studied adsorptive process. The positive value of entropy variation is associated with a decrease in free spaces at the interface liquid solution during Sc(III) adsorption, suggesting that the studied adsorption is a disordered adsorption [61]. Because the free enthalpy has a value lower than 80 kJ mol^−1^, we can conclude that adsorption is a physico-chemical process [62]. The minimum amount of extra kinetic energy required for Sc(III) ions to be adsorbed on the XAD7HP-TOPO surface is 1.72 kJ mol^−1^.

#### 2.3.5. Regeneration and Reusability Studies, Adsorption-Desorption Cycle

In order to reduce the total cost of the recovery process by adsorption, it is important to be able to reuse the adsorbent material as many times as possible. In this context, it is considered that the economic profitability of an adsorptive process is proportional to the regeneration of used adsorbent material. Desorption studies are used to demonstrate the practical applicability of regeneration and reuse of the adsorptive fixed layer in columns [63,64].

**Figure 9:**
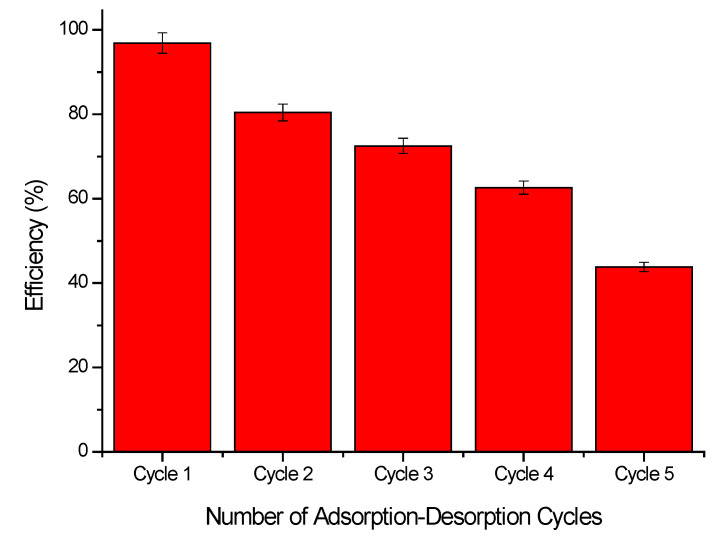
Adsorption-desorption cycle for XAD7HP-TOPO adsorbent material.

In the present study, the Sc(III) ion desorption process has been carried out by treating exhausted XAD7HP-TOPO material with a 5% H2SO4 solution. The obtained experimental data are presented in Figure 9. From these data, we can observe that the newly prepared adsorbent material can be used in five adsorption/desorption cycles. During the adsorption/desorption process, some of the Sc(III) ions are almost irreversibly fixed onto the adsorbent material. Also, as Alsawy et al. [65] proved, a high desorption rate is not automatically translated into high regeneration performance. Such loss of adsorbent performance can be related to the inevitable loss of active adsorbent sites and pore blocking during the desorption process [65]. In the future, works must develop and propose some different functionalizations in order to allow full chemical regeneration of spent adsorbent material.

## 3. Materials and Method

### 3.1. Preparation of XAD7HP-TOPO Material

To obtain the new adsorbent material, functionalization by impregnation of Amberlite XAD7HP resin was performed using the dry SIR method [66,67,68]. The supplier of the desired pendant group used tri-n-octylphosphine oxide—TOPO.

**Figure 10:**
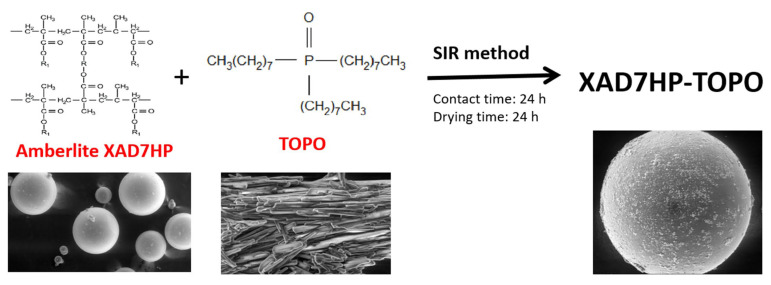
XAD7HP-TOPO obtained using SIR method.

TOPO (Figure 1) is an organophosphorus compound with the formula OP(C_8_H_17_)_3_. It is an air-stable white solid at room temperature [69], which was used as an extraction agent for Sc(III) ions. Amberlite™ XAD™7HP Polymeric Adsorbent (Sigma-Aldrich, Saint Louis, MI, USA) (Figure 10) is a macroporous, non-ionic aliphatic acrylic resin that is supplied as white insoluble beads. Its macroporous structure (containing both a continuous polymer phase and a continuous pore phase), high surface area, and aliphatic nature provide good adsorptive properties [70].

In order to carry out Amberlite impregnation, 1 g of high-purity Amberlite XAD7 (Merck—Sigma Aldrich, Saint Louis, MI, USA) was weighed and further kept in contact for 24 h with 0.1 g of TOPO (an extract) dissolved in ethyl alcohol. After 24 h solvent has been removed by slow evaporation under vacuum. In the next step the impregnated polymer was washed several times with distilled water and dried at 323 °C (preparation steps are presented in Figure 1). To determine the amount of the extractant that was impregnated on the polymeric support, a specific amount of filtered solution was taken before the washing the impregnated polymer, and this solution has been titrated with 0.1 M NaOH. Based on that, it was determined that after impregnation, the newly produced adsorbent material contained about 0.2–0.3 g TOPO per g of XAD7HP [37,40].

### 3.2. Characterization Techniques

After preparation, the newly obtained adsorbent material was characterized using physico-chemical techniques in order to highlight the presence of TOPO-specific groups on the XAD7HP polymeric support. In order to achieve this purpose, the following were used: scanning electron microscopy (SEM) coupled with energy dispersive X-ray spectroscopy (EDX), by using the Quanta FEG 250 scanning electron microscope (FEI, Hilsboro, OR, USA), and Fourier transform infrared spectroscopy (FT-IR), into the spectral range 4000–500 cm^−1^, by using a Shimazdu Ft-IR spectrometer (Shimazdu Corporation, Kyoto, Japan). The specific surface was determined by using the BET method with the Quantachrome Nova 1200e system (Quantachrome Instruments, Boynton Beach, FL, USA), which is based on the measurement of the amount of a specific gas (usually N_2_) adsorbed or desorbed into the analyzed solid. During adsorption or desorption, gas pressure changes until an equilibrium is established.

The adsorptive properties of the material studied are affected, among other things, by the pH of the environment in which it is immersed. In order to establish the influence of pH on the behavior of the adsorbent material in electrolytic media, it is necessary to determine its zero charge point (PZC). Points of zero charge represent the pH value at which the surface of an adsorbent material immersed in a liquid is in equilibrium and has a net electric charge of zero, denoted as pHpzc. The isoelectric point, like the point of zero charge, represents the pH at which the surface has a zero electric charge, but the ions that determine the interface potential are other than H^+^ and OH^-^ ions. When an adsorbent solid is immersed in an electrolytic solution, its surface is electrically charged as a result of processes of dissociation of superficial hydroxyl groups and complexation of some ions from the electrolytic medium, respectively, protonation–deprotonation processes.

The point of zero charge of any adsorbent material can be evaluated by using different specific methods, such as the potentiometric titration method or the equilibrium bringing method (also known as the pH drift method or drift equilibrium) [71,72]. In the present study, the equilibrium-bringing method was used. This method is based on the idea that protons and hydroxyl ions are the ions that determine the potential of the adsorbent surface. In order to determine the zero charge potential of an adsorbent material, follow these steps:

1. Preparation of an initial solution of 0.01 M KCl (Merck—Sigma Aldrich, Saint Louis, MI, USA).
2. Adjustment of the solution pH by using 0.5 M HCl (Carl Roth, Karlsruhe, Germany) or 0.5 M NaOH (Merck, Sigma Aldrich), in the range 2 to 14. Solution pH was measured using a Mettler Toledo Seven Compact—S210 pH meter, with the initial pH being denoted as pH_i_.
3. Solid adsorbent material is mixed with a strong electrolyte solution (KCl) with known pH and concentration in a well-determined and constant solid:liquid ratio. In the present study, 0.1 g of XAD7-TOPO were weighed and placed in contact with 25 mL of electrolyte solution.
4. Obtained suspension was shaken for 60 min by using a Julabo thermostatic bath, and after that, the solution pH was measured, with this pH being denoted as pH_f_.
5. In the last step, we plotted the dependence between pH_i_ and pH_f_. From this dependence, the material zero charge point can be determined as the intersection of this graph with the abscissa.

### 3.3. Sc(III) Adsorption Experiments

In order to determine the optimal dose of adsorbent, the amount of the newly prepared adsorbent material was varied in the range of 0.025 to 0.3 g. In all studied cases, XAD7HP-TOPO adsorbent material was dispersed in a 25 mL solution with an initial concentration of 100 mg Sc(III) per liter and kept in contact for 60 min at 298 K. This solution was prepared by proper dilution starting from the ICP-MS Sc(NO_3_)_3_ standard solution with a concentration of 1000 mg L^−1^(Merck, Sigma Aldrich). Sc(III) residual concentration was determined by using the ICP-MS technique (ICP-MS Plasma Quant 9100, Analytic Jena, Juna, Germany).

To establish the optimal pH for Sc(III) ion recovery, the initial pH of the solutions was varied in the range of 1 to 5. In all studied cases, the following were kept constant: the adsorbent amount (0.1 g), the contact time (60 min), the working temperature (298 K), and Sc(III) ion concentration (100 mg L^−1^). In order to establish the optimal contact time and temperature, the contact time was varied in the range of 15 to 120 min at 4 different temperatures (298, 308, 318, and 328 K). In all these cases, the adsorbent amount was kept constant (0.1 g), the initial solution volume was kept constant (25 mL), and SC(III) ions was 100 mg L^−1^. Based on all these dependencies, the kinetics of the studied adsorption process were established.

In order to establish the condition under which the process equilibrium is established, we used the information obtained from the above experiments and varied only the Sc(III) ion initial concentration into the range of 15 to 200 mg L^−1^. In all these experiments, the dose of the adsorbent material was kept constant at 0.1 g by using 25 mL of Sc(III) solution and a contact time of 90 min and 298 K. In order to highlight the feasibility of the new produced adsorbent material for Sc(III) recovery were performed adsorption/desorption cycles. During these studies, the initial concentration of Sc(III) ions was 160 mg L-1 (the equilibrium concentration established via the equilibrium studies), and the desorption studies were carried out using 5% H_2_SO_4_. All adsorption/desorption studies were carried out using a column with a diameter of 2.5 cm and a height of 30 cm filled with 5 g of adsorbent material. The volume of solution passed through this column was 600 mL, with a flow rate of 10 mL min^−1^.

### 3.4. Determination of Sc(III) Adsorption Characteristics

Adsorption efficiency was determined by using the following equation:

$$\eta =\frac{\left(C_{0}-C_{e}\right)}{C_{0}}100,(\%)$$

and the equation that allows us to determine material adsorption capacity is:

$$q_{e}=\frac{\left(C_{0}-C_{e}\right)V}{m}(\mathrm{mg}/g)$$

where *q_e_*—adsorption capacity, mg g^−1^; C_0_—initial concentration of Sc(III), mg L^−1^; C_e_—residual concentration of Sc(III), mg L^−1^; V—solution volume, mL; m—adsorbent mass, g.

### 3.5. Kinetic Models

Kinetics of the studied adsorptive processes are described by the linear form of the pseudo-first-order [73] and pseudo-second-order [74] models:

ln(*q*_*e*_ − *q*_*t*_) = ln *q*_*e*_ − *k*_1_t

$$\frac{t}{q_{t}}=\frac{1}{k_{2}q_{e}^{2}}+\frac{1}{q_{e}}t$$

where *q_e_* and q_t_ (in mg g^−1^) are the adsorption capacities at equilibrium and time t, respectively, and k_1_ (min^−1^) and k_2_ (in g mg^−1^min^−1^) are kinetic model constants.

The linear forms of the kinetic models were depicted by using the graphical representations ln (*q_e_* − *q_t_*) vs. t and t/*q_t_* vs. t.

### 3.6. Intraparticle Diffusion

It is well known that the adsorption process on porous materials can be described by a complex mechanism consisting of the following three consecutive stages:

1. The transport of ions whose adsorption is studied from the volume of the liquid to the level of the adsorbent material/solution interface.
2. Adsorbate transport from the interface level inside adsorbent material pores (intraparticle diffusion).
3. Retention of the adsorbate ions inside the pores of the adsorbent material by physical or physico-chemical adsorption [75,76].

To distinguish whether film diffusion or intraparticle diffusion is the rate-determining step, obtained experimental data were modeled using the Weber and Morris model:

$$q_{t}=k_{\mathrm{dif}}\cdot t^{0.5}+C$$

where *q_t_*—adsorption capacity at t contact time; k_dif_—the rate constant of intraparticle diffusion (mg/g ∙min^−0.5^); C—a constant correlated with the thickness of the liquid film surrounding the adsorbent particles.

If the dependence between q_t_ and t^0.5^ is a curve with as good a linearity as possible, passing through the origin (C = 0) can say that the intraparticle process is taking place in a single rate-determining step. Otherwise, both intraparticle diffusion and film diffusion influence the adsorption kinetics. A negative value of C indicates that the diffusion film also influences the kinetics of the studied adsorptive process.

### 3.7. Adsorption Equilibrium

Adsorption equilibrium studies were carried out by modeling obtained experimental data with Langmuir [77] and Freundlich [78] isotherms. Linear form of these isotherms is described by the following equations:

$$\frac{C_{e}}{q_{e}}=\frac{1}{q_{m}k_{L}}+\frac{C_{e}}{q_{m}}$$

$$\log q_{e}=\log k_{F}-\frac{1}{n}{\mathrm{logC}}_{e}$$

where *q_m_* (mg/g) is the theoretical adsorption capacity, *k*_L_ and *k*_F_ [(L/g)/(mg/g)1/n] are the Langmuir and Freundlich constants, respectively, and 1/n is the sorption intensity that indicates how beneficial an adsorption is.

Parameters associated with these two isotherms were evaluated from the linear dependences C_e_/*q_e_* vs. C_e_ and log q_e_ vs. logC_e_.

Another isotherm used to characterize the adsorption equilibrium is represented by Sips isotherm [79], which is a combination of the Langmuir and Freundlich isotherms. The nonlinear form of this isotherm is expressed by the following equation:

$$q_{e}=\frac{q_{S}k_{S}C_{e}^{1/n_{S}}}{1+k_{S}C_{e}^{1/n_{S}}}$$

where *q*_S_ is the maximum adsorption capacity (mg/g); C_e_ is the concentration of adsorbate at equilibrium (mg/L); *k*_S_ is a constant related to the adsorption capacity of the adsorbent; n_S_ is a heterogeneity factor.

### 3.8. Adsorption Thermodynamics

A possible adsorption mechanism can be proposed based on the values obtained for Gibbs free energy (ΔG^0^), which can be evaluated based on Gibbs-Helmholtz equation [80]:

ΔG°=ΔH°−TΔS°

where ΔG^0^—Gibbs free energy variation (kJ mol^−1^); ΔH^0^—enthalpy standard variation (kJ mol^−1^) ΔS^0^—entropy standard variation (J mol^−1^·K^−1^); T—absolute temperature (K).

In order to be able to determine the value of Gibbs free energy, we evaluated the values of enthalpy and entropy standard variations from the van’t Hoff equation [80]:

$$\ln K_{d}=\frac{\Delta S{}^{\circ}}{R}-\frac{\Delta H{}^{\circ}}{RT}$$

where: K_d_—equilibrium constant; ΔS°—entropy standard variation (J mol^−1^·K^−1^); ΔH°—enthalpy standard variation (kJ mol^−1^); *T*—absolute temperature (K); *R*—ideal gas constant (8.314 J mol^−1^∙K^−1^).

Information about the way in which the adsorption process is taking place (physical or chemical) can be obtained from the value of the activation energy (Ea). These parameter values are determined by applying the Arrhenius equation [80] to the studied adsorption:

$$k=Ae^{\frac{-Ea}{RT}}$$

where: *k* is speed/rate constant; *T* is absolute temperature (K); *A* is a pre-exponential factor, constant for each chemical reaction; according to the theory of ideal collisions, *A* is the frequency with which collisions with optimal orientation occur; *E_a_* is the activation energy (J/mol); *R* is the universal ideal gas constant (8.314 J/mol K). Activation energy value was determined from the slope of the linear dependence between ln *k*_2_ and 1/*T*.

## 4. Conclusions

The development of new technologies is linked with the usage of different rare earth metals, among which a special place is occupied by Sc ions. Keeping this in mind, the presence of Sc(III) ions in different industrial wastes transforms them into possible raw materials for Sc production.

A new adsorbent material has been prepared by functionalization through the impregnation of a solid support using the SIR method. Amberlite XAD7HP was used as solid support, while extractant TOPO was used. The functionalization process was carried out under the following conditions: support: extractant ratio = 10:1, a contact time of 24 h, and a 24 h drying time. After this step, the successful functionalization of Amberlite resin was proven by SEM, EDX, and FT-IR characterization. Concomitantly, the decrease in the specific surface of the newly prepared adsorbent material in comparison with the one of the non-functionalized Amberlite resin proves the successful implementation of the functionalization process.

In case of studied Sc(III) ions recovery was obtained a maximum adsorption capacity of 31.84 mg Sc(III) ions per gram of adsorbent material. This maximum value has been obtained by using the following conditions: pH > 3, a solid:liquid ratio of 0.1 g adsorbent material at 25 mL of Sc(III) solution, a contact time of 90 min, 298 K, and a Sc(III) ion initial concentration equal to 160 mg L−1.

By modeling the obtained experimental data, it was proved that the Sc(III) adsorption is described by a pseudo-second-order kinetic model. Also, it was determined that the main mechanism involved in Sc(III) adsorption is represented by the formation of chemical bonds between Sc(III) ions and adsorbent superficial active centers. By modeling experimental data with an intraparticle diffusion model, it was evidenced that the adsorptive process is taking place in two distinctive stages. Studied Sc(III) ion recovery is better described by the Sips isotherm, with multilayer adsorption capacity increasing with temperature increase. Thermodynamic studies have evidenced that the free Gibbs energy variation has negative values, meaning that the studied adsorptive process is a spontaneous one. Positive values of enthalpy variation confirm the endothermal nature of the studied process at the considered temperature interval. In order to prove the feasibility of a newly prepared adsorbent material for Sc(III) ion recovery, adsorption and desorption studies were carried out, which revealed that XAD7HP-TOPO can be used for five adsorption/desorption cycles.

## Figures and Tables

**Table 1:** Kinetic parameters for the adsorption of Sc(III) onto XAD7HP-TOPO.

Pseudo-First-Order					
Temperature (K)	*q*_e,exp_ (mg g^−1^)	*k*_1_ (min^−1^)	*q*_e,calc_ (mg g^−1^)	*R* ^2^	$\bar{R^{2}}$
298	20.24	0.0168	20.61	0.9715	0.8210
308	21.20	0.0174	18.58	0.9247	0.7456
318	22.24	0.0209	15.72	0.9253	0.7605
328	23.03	0.0268	16.53	0.9333	0.7792
Pseudo-second-order					
Temperature (K)	*q*_e,exp_ (mg g^−1^)	*k*_2_ (g mg^−1^∙min^−1^)	*q*_e,calc_ (mg g^−1^)	*R* ^2^	$\bar{R^{2}}$
298	20.24	390.8	25.12	0.9933	0.9875
308	21.20	498.4	25.51	0.9901	0.9884
318	22.24	697.5	25.70	0.9943	0.9949
328	23.03	882.6	25.83	0.9943	0.9953
Intraparticle diffusion model (IPD)					
Temperature (K)	K_diff_ (mg·g^−1^ min^−1/2^)	C		*R* ^2^	$\bar{R^{2}}$
298	1.17	4.02		0.9528	0.8112
308	1.78	5.39		0.9093	0.7635
318	2.16	6.31		0.8603	0.7504
328	2.28	7.56		0.8403	0.7425

**Table 2:** Parameters of adsorption isotherms for adsorption of Sc(III) onto XAD7HP-TOPO.

Langmuir Isotherm			
*q*_m,exp_ (mg/g)	*K*_L_ (L/mg)	*q*_L_ (mg/g)	*R* ^2^
31.84	0.112	38.14	0.9849
Freundlich isotherm			
*K*_F_ (mg/g)	1/*n*_F_		*R* ^2^
7.91	0.36		0.8880
Sips isotherm			
*K*_S_	*q*_S_ (mg/g)	1/*n*_S_	*R* ^2^
0.075	34.6	0.04	0.9915

**Table 3:** Comparison with the literature.

Materials	Sc(III) Recovery Efficiency, %	References
732-type acid cation exchange resin	84.2	[56]
TP 272-Cyanex 272	-	[57]
Amberchrom CG-71c nonionic macroporous sorbent	-	[58]
polymer support fabric (PP-g-PGMA)	98	[59]
glycol amic acid embedded resin	45	[60]
XAD7HP-TOPO	99.7	Present paper

**Table 4:** Thermodynamic parameters for adsorption of Sc(III) onto XAD7HP-TOPO adsorbent material.

ΔH^0^ (kJ/mol)	ΔS^0^ (J/mol∙K)	ΔG^0^ (kJ/mol)				*R* ^2^
26.28	88.53	298 K	308 K	318 K	328K	0.9917
		−26.35	−27.24	−28.12	−29.01	

## Data Availability

Data are contained within the article.
